# The Story of the Insane from Year to Year

**Published:** 1903-08-29

**Authors:** 


### THE STORY OF THE INSANE FROM YEAR TO YEAR.

### Fifteenth Annual Series.

### LONDON COUNTY ASYLUM AT BEXLEY.

On the first of January this asylum contained 1,425 patients, and on December 31st the number had risen to 2,051. There were 1,145 first admissions, 30 readmissions, 202 discharged recovered, 32 discharged relieved, and 281 died. The total number under treatment was 2,600, and the average daily number resident was 1,893. Excluding transfers, the recoveries stand at 27·41 per cent. of the admissions, and the deaths stand at 14·84 per cent. of the average number resident. The first of these percentages is low and the second is high. Of the year's deaths 126 were caused by cerebral and spinal diseases (general paralysis being responsible for 89), 25 by consumption, 35 by other lung diseases, and 15 by colitis. Of the year's admissions 130 were supposed to have their insanity caused by such moral causes as domestic trouble, anxiety, religion and fright; while of the physical causes, alcohol accounts for 181 and syphilis for 195. Hereditary influence does not appear as a cause, and this is about the first time we have ever known it absent in the admissions of a county asylum. One of the leading features of this asylum is that many of the patients are accommodated in villas; and Dr. Stansfield says he has "no hesitation in pronouncing in the strongest terms in favour of it." As the more general barrack system is also typified at Bexley Heath, it may be taken that he is well qualified to express an opinion, and that this opinion will carry due weight. On the great drink question Dr. Stansfield reminds us that "there is a very common fallacy prevalent among the laity that a man or woman cannot be suffering from the effects of alcohol unless he or she has been in the habit of taking sufficient at one time to produce alcoholic poisoning, whereas these form only a very small percentage; the bulk of cases admitted into asylums as the victims of alcohol are people who have long been in the habit of 'nipping,' a habit very often indulged in secretly, particularly by women." Of severe diarrhœa and dysentery (or colitis) there were 129 cases and 15 deaths. Dr. Stansfield is satisfied that it was brought to his asylum by cases transferred from other asylums, and indeed there would seem to be no escape from this conclusion, for "insanitary conditions and over-crowding, the conditions supposed specially to favour colitis and dysentery, have never obtained here. The drainage is on the most modern principle, with automatic flushing tanks which are timed to go off once in 48 hours with a discharge of 3,144 gallons, and is connected with the local sewage system." In spite of all this it is feared that this asylum scourge has become endmic at Bexley Heath, although every case of diarrhœa is treated as if it were infective, and isolation is continued for 14 days after the symptoms have passed off. The report is an able and interesting one. When speaking of criminal lunatics, Dr. Stansfield says truly that "the whole of the teaching in a modern asylum aims at the elimination of the prison element in housing and dealing with the insane, whilst the compulsory association of the non-criminal with the criminal class is a gross injustice to the former." So it undoubtedly is; and so medical superintendents have pointed out until they must be well-nigh tired of the subject. In fact our Lunacy Laws need a searching revision, but not on the lines of the last Act, which could hardly be said to satisfy anyone. A very important part of the administration of an asylum is that of maintaining the air in the wards at a suitable temperature; and we have more than once emphasised the fact that this can only be done satisfactorily by open fireplaces, although this old system may occasionally be helped by other means when the temperature is very low. We do not know how Bexley Heath Asylum is warmed, but we come across the following sentence: "The system of open pipes and radiators in the wards and passages is certainly efficient as regards the heating and economy, but it has so many disadvantages from dangers, dirt and noise as to preclude its adoption in these institutions." In spite of such warnings, vast sums are being spent in our county asylums on one or other of these pernicious systems of heated air. The Committee express their appreciation "of the zeal and efficiency with which Dr. Stansfield, his medical colleagues and the staff generally continue to discharge their duties." The weekly cost per head is about 10s. 9d.

### GARTLOCH ASYLUM, GLASGOW.

THE names of 539 patients were on the books at the beginning of the year, and this number had increased to 587 at the end of the year. There were 174 first admissions, 79 readmissions, 107 discharged recovered, 26 discharged relieved, 29 discharged not improved, and 52 deaths. The average number resident was 567, and the total number under treatment during the year was 796. Of the year's deaths 24 were caused by cerebral and spinal diseases, 9 by consumption, 4 by other lung diseases, and 1 by colitis. Of the year's admissions 13 were supposed to owe their insanity to moral causes. Alcohol accounts for 89, and heredity for 9; but there was a history of alcoholism in 10 additional cases, and there was hereditary predisposition to drink in other instances. Although only 9 deaths were certifiable to consumption, it was shown by the post-mortem examinations that active tubercular disease had existed in 35 per cent., and quiescent tubercular lesion in 19 per cent.—that is, of the post-mortems. In many of the Scottish asylums much personal freedom is afforded the patients, and we note with pleasure that at Gartloch 83 men and 54 women are on parole, and of these only 9 broke their parole during the year. In addition to Dr. Parker, the recently-appointed medical superintendent, there are three resident assistant medical officers, and one of these is a lady. Clinical assistants are also employed in the wards, so that the medical element is strong in this asylum.

